# Supplementary material for: Development of a Duplex Serological Multiplex Assay for the Simultaneous Detection of Epstein-Barr Virus IgA and IgG Antibodies in Nasopharyngeal Carcinoma Patients
Source: Cancers (Basel). 2023 Apr 30;15(9):2578. doi: 10.3390/cancers15092578 (PMC10177259; doi:10.3390/cancers15092578)
Supplement: Supplementary file 1 [file cancers-15-02578-s001.zip › cancers-2156127-supplementary.pdf]

<u>Supplement</u>

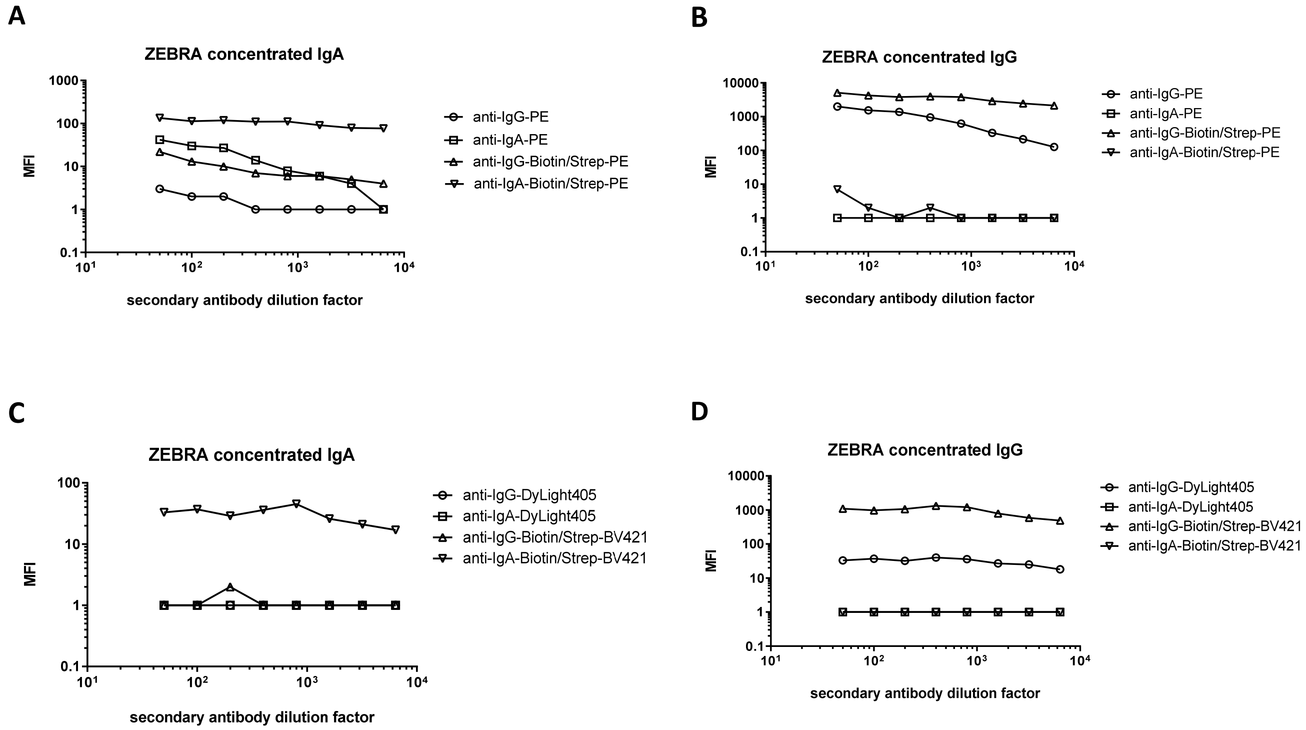

Supplementary Figure S1: Evaluation of specific secondary antibodies in combination with various fluorescent dyes. *Data of one antigen (ZEBRA) is shown exemplarily. A: Directly labeled and biotinylated secondary antibodies with PE tested against concentrated IgA antibodies. B: Directly labeled and biotinylated secondary antibodies with PE tested against concentrated IgG antibodies. C: Directly labeled and biotinylated secondary antibodies with violet dyes tested against concentrated IgA antibodies. D: Directly labeled and biotinylated secondary antibodies with violet dyes tested against concentrated IgG antibodies. MFI: median fluorescence intensity.*

Supplementary Table S1. Cut-off values for EBV IgA and IgG antibodies according to EBER-ISH status (based on ROC analysis with ≥ 90% specificity) for 41 NPC tumors (29 positive, 12 negative).

Antigen	Cut-off			Sensitivity			Specificity		
	Luminex	BV-Duplex	SB-Duplex	Luminex	BV-Duplex	SB-Duplex	Luminex	BV-Duplex	SB-Duplex
BFRF1 IgG	30*	32	42	79.3	79.3	79.3	91.7	91.7	91.7
BGLF2 IgG	241	251	271	96.6	100	100	91.7	91.7	91.7
BORF1 IgG	93	570	506	37.9	24.1	27.6	91.7	91.7	91.7
BPLF1 IgG	141	565	702	65.5	28.6	55.2	91.7	91.7	91.7
BRLF1 IgG	38	30*	30*	82.8	86.2	86.2	91.7	100	91.7
BXLF1 IgG	204	447	510	93.1	96.6	96.6	91.7	91.7	91.7
BZLF1	32	92	88	51.7	86.2	93.1	100	91.7	91.7
EA-D IgG	851	700	614	89.7	89.7	96.6	91.7	91.7	91.7
EBNA1pep IgG	3589	5142	5295	44.8	58.6	58.6	91.7	91.7	91.7
EBNA1trunc IgG	4203	3741	3993	89.7	72.4	72.4	91.7	91.7	91.7
LF2 IgG	30*	30*	31	96.6	96.6	96.6	100	100	100
VCAp18 IgG	8171	8984	8438	6.9	13.8	17.24	91.7	91.7	91.7
ZEBRA IgG	727	1233	1195	82.8	79.3	86.2	91.7	91.7	91.7
BFRF1 IgA	52	30*	30*	86.2	69	69	91.7	100	100
BGLF2 IgA	2158	134	153	75.9	82.8	82.8	91.7	91.7	91.7
BORF1 IgA	156	30*	30*	44.8	41.4	44.8	91.7	91.7	91.7
BPLF1 IgA	894	197	397	44.8	44.8	41.4	91.7	91.7	91.7
BRLF1 IgA	155	30*	30*	72.4	65.5	69	91.7	91.7	91.7
BXLF1 IgA	1367	86	95	79.3	86.2	82.8	91.7	91.7	91.7
BZLF1 IgA	30*	30*	30*	48.3	48.3	51.7	91.7	91.7	91.7
EA-D IgA	3968	213	248	69	89.7	89.7	91.7	91.7	91.7
EBNA1pep IgA	453	151	150	58.6	55.2	62.1	91.7	91.7	91.7
EBNA1trunc IgA	477	86	93	79.3	89.7	89.7	91.7	91.7	100
LF2 IgA	30*	30*	30*	93.1	69	72.4	91.7	100	100
VCAp18 IgA	5123	678	658	65.5	58.6	62.1	91.7	91.7	91.7
ZEBRA IgA	1303	114	120	55.2	62.1	65.6	91.7	91.7	91.7

* Lower limit of quantification (LLOQ) below 30 MFI
